# 

**Published:** 1917-01-27

**Authors:** 


### CONTENTS.

PAGE

**Dishonesty in Testimonials...** ... ... ... ... 331

**Maternity and Child Welfare** ... ... ... 332

**Hospital and Institutional News ...** ... ... 333

The King and the London Hospital Saturday Fund—King Manoel and Military Orthopædics—Shortage of Residents in Hospitals—A Hospital Not a "National Industry"—The Home at Richmond Hill—A Miners' Mistake in South Wales—Can a Hospital Accept Conditional Contributions?—A Handsome Library for Patients—A Symbol of Growth—The Need for Medical Assessors in Compensation Litigation—An Ad Misericordiam Appeal Successful—The New Government and Infant Welfare—Miners' Subscriptions Doubled—Developments at Letterkenny—Death of Miss Mary Wardell—Dumfries Parish Council and the Moorheads Hospital—Irregularities at the West Riding Asylum—The St. John Ambulance Brigade Hospital—Clearing House for Organisers—Precautions in Theatre Work—The Smallpox Peril—Health Visitors' Salaries—A Strict Disciplinarian—Infant Welfare in Cornwall—The Supply of Nurses in Wales—A Pharmacists' Field Ambulance—This Week's Drug Market.

**The Johns Hopkins Hospital, Baltimore** ... 339

Unique, Up to Date, and Inspiring.

**Reform in Tuberculosis Work** ... ... ... 341

III. The Tuberculosis Officer.

**On the Night of the Explosion** ... ... ... 343

How the London Hospital Organised and Suffered. The Explosion on January 19.

PAGE

**The Matrons' and Sisters' Department** ... 345

Enter the Royal British College of Nursing. Round the Hospitals. All that Happened at R.B.N.A. Meeting—The Way to the Money—Some Splendid Speeches at Leeds—Lay Participation Demonstrates its Value—Ireland Next.

**The Royal British College of Nursing ...** ... 348

Important Meeting at Leeds: Large Funds Forthcoming.

**The Editor's Letter-Box** ... ... ... ... ... 349

Churches and Charity Funds—Deaths from Fire: Are Trained Nurses' Uniform Dangerous?

**The "Mobilisation" of the Medical Profession** 350

(From the National Medical Union.)

**The Royal British Nurses' Association** ... 351

The Royal British College of Nursing Born.

**Medical Appointments** ... ... ... ... 354

**Matrons' and Sisters' Appointments** ... ... iv

**Gifts and Bequests** ... ... ... ... ... iv

**Institutional Needs** ... ... ... ... ... iv

**Passing Events and Topics...** ... ... ... iv

**The Institutional Worker** ... ... (Suppl.) 1

Precautions in Theatre Work at Operations. Questions for January. Enquiries and Answers. The Sick and in Need. Miscellaneous.

